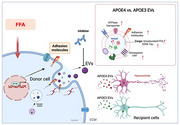# APOE4 drives neuroinflammation and lipid dysbiosis in Alzheimer's disease by modulating lipid compositions and cell adhesion molecules in brain‐derived extracellular vesicles

**DOI:** 10.1002/alz70855_105076

**Published:** 2025-12-24

**Authors:** Zhengrong Zhang, Kaiwen Yu, Hanmei Bao, Tadafumi C Ikezu, Arun Reddy Ravula, Bridgette Melvin, Clara Scholes, Yang You, Justice Ellison, Takahisa Kanekiyo, Michael A. DeTure, Dennis W. Dickson, Xianlin Han, Junmin Peng, Seiko Ikezu, Tsuneya Ikezu

**Affiliations:** ^1^ Mayo Clinic Florida, Jacksonville, FL, USA; ^2^ St. Jude Children's Research Hospital, Memphis, TN, USA; ^3^ University of Texas Health Science Center at San Antonio, San Antonio, TX, USA; ^4^ Mayo Clinic, Jacksonville, FL, USA; ^5^ University of Texas Health Sciences Center, San Antonio, San Antonio, TX, USA

## Abstract

**Background:**

Extracellular vesicles (EVs) are key mediators in transferring pathological proteins associated with Alzheimer's disease (AD). The apolipoprotein E (APOE) gene, particularly the ε4 allele, is a major genetic risk factor for late‐onset AD. However, the influence of APOE genotype on the biological characteristics and cargo composition of brain‐derived extracellular vesicles (BDEVs) in AD remains poorly understood.

**Method:**

In this study, BDEVs were isolated from human AD temporal cortex with APOE3/3 and APOE4/4 genotypes (*N* = 20 / group). Nanoflow cytometry and super‐resolution microscopy were used to evaluate the tau load within these BDEVs. EV‐mediated tau transmission was assessed in aged human Tau KI and APP^NL‐G‐F^: Tau KI mice *in vivo*. BDEV uptake and tau seeding were evaluated in iPSC‐derived neurons through live imaging. Additionally, shotgun lipidomics and data independent acquisition proteomics were analyzed lipidome and proteome of BDEVs, respectively. Key molecules linked to APOE genotype were identified through integrated weighted gene co‐expression network and trait correlation analysis.

**Result:**

APOE4/4 BDEVs exhibited a higher proportion of phosphorylated tau (pS396) at single EV levels. Upon injection of 300pg tau of BDEVs into aged Tau KI and APP^NL‐G‐F^: Tau KI mice, APOE4/4 BDEVs significantly increased AT8^+^ phosphorylated tau levels, and induced neuroinflammation *in vivo*. Moreover, APOE4/4 BDEVs demonstrated stronger neuronal uptake and tau transfer compared to APOE3/3 BDEVs, impairing the neuronal activity of iPSC‐derived neurons. Lipidomic revealed that APOE4/4 BDEVs were significantly more enriched in pro‐inflammatory long‐chain polyunsaturated fatty acids (PUFA) compared to APOE3/3 with Braak stage association. Furthermore, gene ontology pathway analysis using differentially expressed proteins in APOE4/4 BDEVs showed increased activity in oxidative phosphorylation and sterol‐binding. Multi‐omics integrated data revealed that upregulation of PUFA was significantly associated with elevated cell adhesion molecules and ATPase transporter activity with neural cell adhesion molecule as one of key molecule. Notably, neutralizing antibody against the molecule effectively reduced APOE4/4 BDEV internalization and mitigated EV‐mediated tau pathology in recipient cells.

**Conclusion:**

Our comprehensive analysis suggested that AD APOE4/4 BDEV may enhance transmissibility of tau pathology and neuroinflammation via enriched pro‐inflammatory PUFA and cell adhesion molecules. Targeting these pathways presents a promising therapeutic strategy to mitigate disease progression, particularly in APOE4 individuals.